# Analysis of protein interaction network in obesity linked with periodontitis

**DOI:** 10.6026/9732063002001683

**Published:** 2024-12-31

**Authors:** Vinayagam Ramya, Amaldas Julius, Jagadeesan Bhuvaneswarri, Padmanaban Preethi, Farjana Nilofer, Fiona Angelin

**Affiliations:** 1 Department of periodontology and implantology, Sree balaji Dental College and Hospital, Biher university, Chennai, India; 2 Department of biochemistry, Sree balaji Dental College and Hospital, Biher university, Chennai, India

**Keywords:** Obesity, periodontitis, periodontal disease, protein-protein network interaction, gene ontology, bioinformatics

## Abstract

The clinical link between obesity and periodontitis by investigating the potential interaction among proteins is of interest. A protein-protein interaction (PPI) network analysis using STRING was conducted on novel proteins identified from saliva samples through quantitative proteomics and mass spectrometry. Twenty proteins were involved in molecular process, cellular components and biological functions focusing on proteins with variants closely associated with obesity and periodontitis. Analysis revealed that PPI network exhibited a high degree of interactions, indicating a biological connection with a significant p value 4.8*105. These PPIs discuss the interplay between obesity and periodontitis, require further experimental validation, unravel new clues for downstream studies and propose biological mechanisms through which these two conditions may interact.

## Background:

Periodontitis is a polymicrobial disease caused by the development of bacterial biofilms. It is a complex and heterogeneous oral cavity disease [1]. The initiation of a chronic inflammatory response in periodontal tissues leads to a microbial shift favouring pathogenic microflora [2]. It has been shown that periodontitis affects a person's risk of developing systemic diseases. Therefore, the search for biological indicators that can be used to estimate, thereby preventing and diagnosing illness in its initial phases, has received significant attention [3]. Obesity, reported as a primary global public health concern, is often characterised by excessive accumulation of body fat and affects millions of individuals worldwide [4]. The prevalence of obesity has been steadily increasing over the past few decades, with more than 800 million adults categorised as obese in 2020 [5]. Periodontitis and obesity are also correlated. Obesity is a chronic low-grade inflammation that leads to the excessive accumulation of adipose tissue and tissue damage [5]. Inflammation weakens the immune system and activates proinflammatory cytokines, intensifying the body's reaction to bacterial infections in periodontitis [6]. Furthermore, obesity increases the likelihood of type 2 diabetes mellitus and causes insulin resistance, both of which are independent risk factors for periodontitis [7]. Proteomics, a large-scale study of proteins, offers a means of comprehending the molecular landscape of obesity by identifying essential proteins and their interactions [8]. It helps capture the fluidity of biochemical pathways in periodontitis. Therefore, it is logical to consider the mechanisms that may contribute to the risk of obesity associated with Periodontitis, as evidence from epidemiology and clinical studies provides insight into their relationship [9]. Proteins are categorised into biological processes, molecular functions and cellular components using gene ontology (GO) enrichment analysis [10]. In contrast, protein-protein interaction (PPI) networks show how proteins operate together in biological systems. Together, these methods provide a better understanding of the molecular causes of obesity [11]. This review focuses on STRING-based PPI network and GO enrichment analysis findings from an obesity study. These findings highlight essential proteins and pathways associated with oxidative stress, immunological responses and metabolic control. STRING Gene Ontologies and Systems Biology Systems biology extends beyond identifying individual proteins to analysing their interactions and interconnections. Protein-protein interaction networks created by bioinformatics tools (*e.g.*, STRING-Search Tool for the Retrieval of Interacting Genes/Proteins) enable us to study how proteins cooperatively work together in complex biological systems [12]. STRING is a functional partnership between proteins based on experimental evidence, computational predictions and scientific text respectively [1]. GO analysis: Gene Ontology enrichment analyses, which categorise proteins according to biological processes, molecular functions and cellular components, were also performed using STRING. GO analysis revealed broader biological roles for the proteins detected in this study. Together, STRING and GO analyses provide a broad picture of how these protein interactions affect the proteome [14]. This review sought to analyse the findings of a STRING-based protein-protein interaction (PPI) network assessment and Gene Ontology enrichment, specifically in obesity. By emphasising the molecular interactions and biological processes identified through these systems biology methodologies, we aimed to elucidate the fundamental mechanisms that contribute to obesity while refraining from a direct discussion of the proteomic profile utilised in the research. Our interpretation of these findings highlights the interconnected networks of proteins associated with metabolism, immune regulation and oxidative stress, which are all vital to the onset and progression of obesity.

## Materials and Methods:

## Data source:

The bioinformatics tool obtained the PPI interaction network of obesity and periodontitis and compared it with the standard STRING database, which has a sub-database of protein interaction resources relevant to the analysed dataset, such as obesity-related proteins.

**Figure 1:**
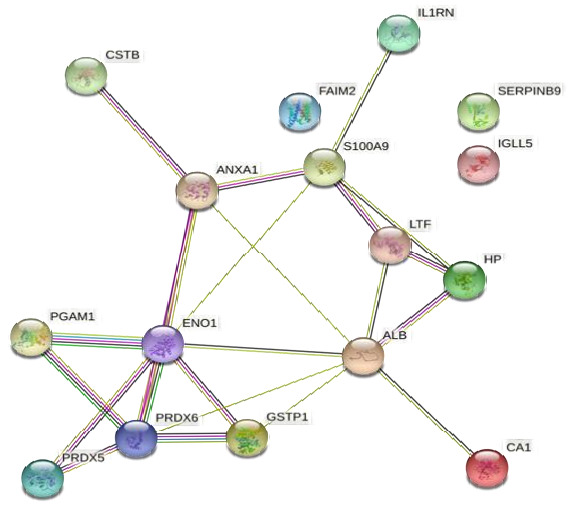
Common PPI network interaction of obesity and periodontitis (string database) Periodontitis (string database)

**Figure 2:**
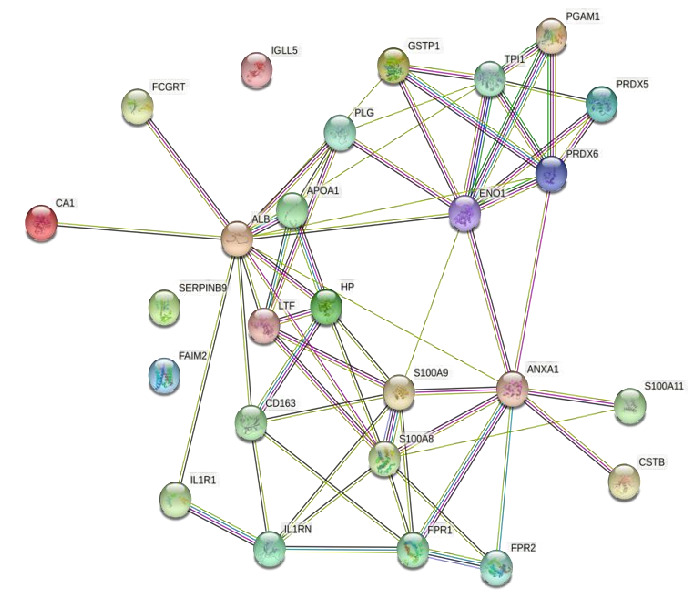
PPI network interactions of obtained proteins

**Figure 3:**
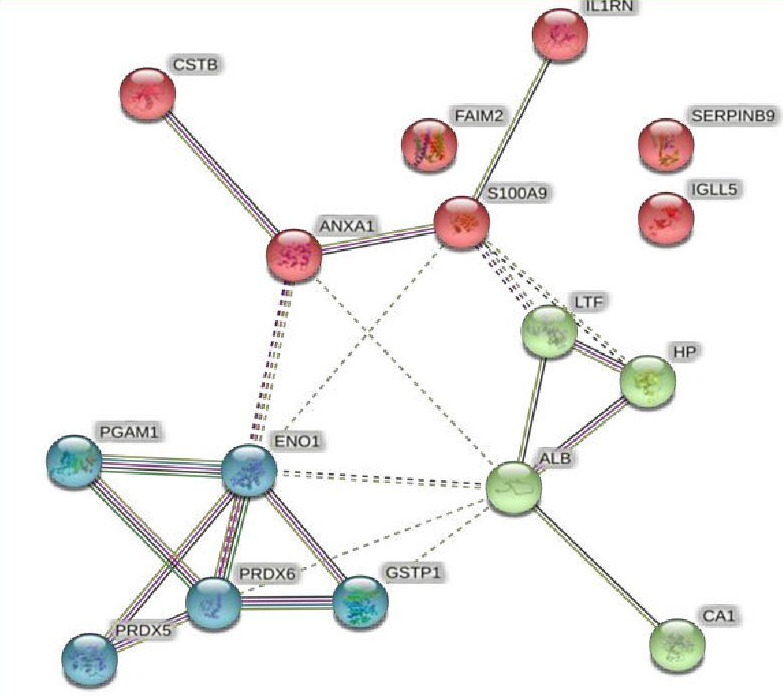
Cluster analysis Core cluster (blue nodes); centered clusters (pink nodes); abn-centered clusters (green nodes); pheriphral cluster (mixed node)

Figure 2 reveals the protein-protein interaction (PPI) from the obtained salivary proteomic profile of 20 proteins Figure 1 Search Tool for the Retrieval of Interacting Proteins (STRING) analysis for common proteins for obesity and periodontitis analyzing the network properties, it was found that the PPI network had more interactions among themselves than expected for a random set of proteins of similar size drawn from the proteome, indicating that the proteins are at least partially biologically connected (p-value = 4.8*10-5). Finally, from the expected number of 13 edges, a final number of 33 edges (average cluster distance = 47.53; average local clustering coefficient = 0.33) were cast. A sensitivity analysis with a confidence cut off of 0.64 revealed that the results were independent of the choice of the confidence cut off since the same network emerged (Figure 1). The nodes represent proteins and the lines represent protein interactions. String analysis identified several essential proteins involved in critical biological processes such as metabolism, immune response and oxidative stress [13] [14]. Double bonds within proteins indicate more robust or supported interactions based on higher confidence levels (Figure 2). ENO1, PRDX5 and PRDX6 indicate that these proteins are closely intertwined within biological systems and likely function together in oxidative stress or metabolic pathways [15]. According to the bond thickness, triple bonds or thicker lines between ENO1, PRDX5, PRDX6, GSTP1 and PGAM suggest that they strongly interact with these proteins as part of a shared, closely related functional network [16] [17]. PRDX5 and PRDX6 (Peroxiredoxins) (Figure 3). These are antioxidants that scavenge lipid peroxides when oxidative stress increases in obesity. Their strong interactions suggest they may function together to control cellular oxidative damage [18] [19]. ENO1 (alpha-enolase) is involved in glycolysis and is an essential component of energy generation. Its intense interaction with peroxiredoxins may reveal a connection between energy metabolism and oxidative stress, a critical component of metabolic dysfunction [20]. PGAM1 is a critical enzyme catalyses the conversion of 3 phosphorylation (3PG) to 2 phosphoglycerate (2PG) during glycolysis. [21] GSTP1-glutathione s TRANSFERASE-they bring about oxidative stress protection [22]. Other proteins seen in this network are FAIM2 -Fas apoptotic inhibitory molecule 2, otherwise called as NMP35 protein or LFG (lifeguard) enables calcium-channel activity, involved in regulation of neuron apoptic process. Annexin (ANXA1) a cytoskeletal protein involved in progression of Periodontitis. SERPIN, Cystatin-B is protease inhibitors helping in regulating proteolytic pathways. LTF, Lactoferrin glycoproteins are secreted higher levels in extracellular matrix during inflammation. IGLL5, lambda-like polypeptide 5 F are immunoglobulins related to immune response [23] [24].

## Results:

The F tests should be used only for descriptive purposes because the clusters have been chosen to maximize the differences among cases in different clusters. (Table 1) The observed significance levels are not corrected for this and thus cannot be interpreted as tests of the hypothesis that the cluster means are equal (Table 2 & Table 3).

## Gene enrichment analysis:

The analysis under the Reactome-Pathway revealed the primary count and association of genes involved in the immune system, cellular stress, metabolism and other essential biological processes. They are involved in glycolysis and gluconeogenesis according to RT (reactions detailed resource link).

## Gene ontology analysis:

**Figure 4:**
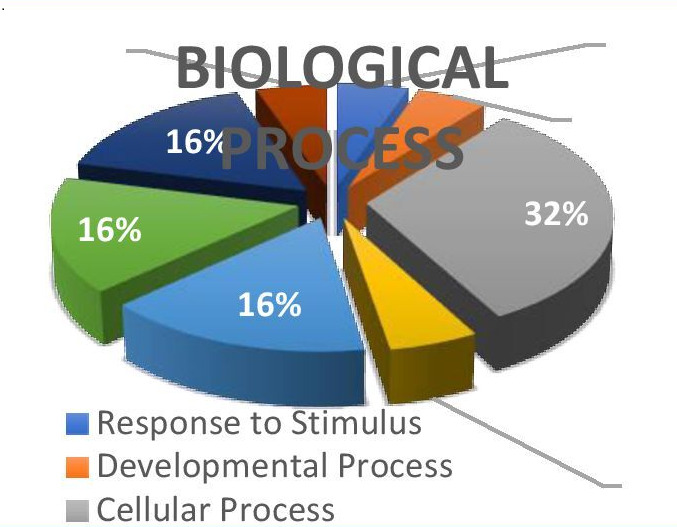
Gene ontology analysis - biological process and protein classes

**Figure 5:**
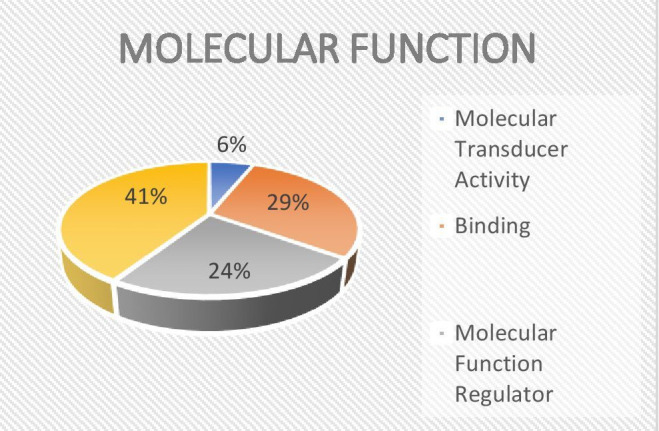
Gene ontology analysis - molecular function

**Figure 6:**
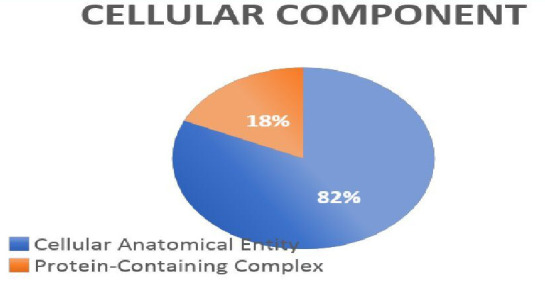
Ontology analysis - cellular component

Using GO annotation, the identified proteins were grouped into the molecular function, cellular component, biological process and Protein Class categories (Figure 4, Figure 5, Figure 6).

## Discussion:

We used a bioinformatic approach to predict a potential PPI network between obesity and periodontitis. Although these PPIs require further experimental validation, they unravel new clues for downstream studies and propose biological mechanisms through which these two conditions may interact. The interaction established by enol1, phosphoglycerate ligase, glutathione S Transferase and peroxiredoxins PRDX5 and PRDX6 is a strong candidate in this study (Table 4), peroxiredoxins are a ubiquitous family of cysteine-dependant peroxidase enzymes usually observed when the PMNS becomes hyperresponsive in the case of any microbial insult and exhibits increased production of reactive oxygen species [25- 26]. Therefore, the antioxidant capacity decreases. Generally, glutathione (GSH) is reduced in the GCF of aggressive and chronic periodontitis patients. Under physiological conditions, GSH blocks the ROS-mediated activation of transcription factor nuclear factor κB (NF-κB) and the subsequent upregulation of proinflammatory cytokine production. These results indicate that GSH is present at higher concentrations in the salivary profile of obese individuals and periodontitis patients, as it has potent anti-inflammatory and antioxidant capacities. ENO1 and PGAM1 are proteins involved in glycolytic, carbohydrate catabolic, glucose metabolic, organophosphate metabolic and carbohydrate-derivative metabolic processes. They are highly expressed in individuals with obesity [27]. Similarly, peroxiredoxins are antioxidants that prevent the oxidation of substrates by Reactive oxygen species (ROS) and offer protection against oxidative stress [28]. In periodontitis, oxidative stress can play a significant role in inflammation and disease progression; it neutralises ROS and stimulates the inflammatory cascade by regulating the production of pro-inflammatory cytokines, especially IL1 β and TNF-α, thereby limiting and creating a favourable environment for the healing of inflamed tissue [29]. Our study's PRDX5 and PRDX6 interactions suggest that oxidative stress is a significant risk factor for obesity.

Increased levels of peroxidases and glutathione S Transferase (GSPT-1) were similar to the findings of a study conducted by Cerzenski *et al.* [15]. In this study, we also analysed the abundance of proteins, such as haptoglobin, S100A9 and Albumin, all previously linked to periodontitis. Enriching immune-related proteins, such as S100A9 and haptoglobin, underscores the role of inflammation in obesity. These proteins are central to the chronic inflammatory state observed in obesity and exacerbate metabolic dysfunctions [30].

Albumin regulates the colloidal osmotic pressure of the blood and hormones and acts as an ion transporter. Some periodontal microbes that trigger an inflammatory response, such as T. denticola, are believed to increase salivary albumin levels [31]. These microbes also use Albumin and Immunoglobulins as their energy sources. S100A9 is a calcium- and zinc-binding protein whose functions include proinflammatory, antimicrobial, oxidant scavenging and apoptosis-inducing activities [32] [33]. In this PPI interaction, S100A9 is expressed when obesity triggers cellular defence mechanisms and response to microbial stimuli to combat oxidative damage and inflammation. Cystatins are protease inhibitors abundantly found in saliva and they play an essential role in inhibiting tissue-destructive proteases in inflammatory processes, such as lysosomal cathepsins B, H and L in the oral cavity [34] [35]. Lactotransferrin is an iron-binding protein and its antibacterial effect is achieved by competing for iron with bacteria, there by inhibiting bacterial growth. Lamy *et al.* (2015) reported higher levels of Zinc-α-2 glycoprotein in individuals with obesity, as well as a tendency for them to present higher levels of Carbonic Anhydrase 6 (CA-VI); our finding is also partially by this study [36]. In the recent years of adipose tissue research, the perception of fatty tissue from merely being a passive lipid storage sign has changed to insights into its crucial role in managing whole body homeostasis, metabolism, inflammation and immune response [37]. It acts through these bioactive molecules called adipokines [38]. A study by Novkovic *et al.* stated that vaspin (SERPIN A12) is expressed and positively correlated with BMI and insulin sensitivity. Similarly, the SERPIN8 interaction in this PPI network may be a response to diminished insulin activity and resistance [39] [40]. Misaki *et al.* stated that phorphyromonas gingivalis-induced endotoxemia alter the endocrine function. The infiltration of immune cells by macrophages results in the abnomal secretion of adipokines leading to enhanced activation of pyrine domain activating protein (NLRP3) INFLAMMASOME and increased susceptibility to infection [41]. Moreover Prx1 eliminates intracellular ROS and exhibits a cytoprotective role in LPS-induced apoptosis. However, under physiological conditions, Prx1 overexpression acts as a H2O2 messenger, triggering the expression of ASK1 and its downstream cascades [41]. Chang *et al.* has suggested that with non-surgical periodontal therapy, Cu/ZnSOD, MnSOD, catalase and Prx2 are significantly decreased [42]. Therefore the combination of obesity and periodontitis exacerbates the inflammation and adipose tissue homeostasis in turn, obesity severes periodontitis with shared molecular signatures [43].

## Conclusion:

The core clusters reflect the plasma proteins associated with immuno-modulatory responses and the centred clusters of plasma proteins demonstrate the role of proteins involved in maintaining homeostasis, anti-oxidant defence and ion metabolism. Furthermore, network analysis revealed essential hub proteins such as (ENOL1, PGAM, GSPT, PRDX5 and PRDX6), which play a pivotal role in the cross-talk between PD and its comorbidities, offering potential targets for therapeutic intervention. Therefore, this study identified proteins that are potential key players in biological processes and inflammation.

## Funding:

This study did not receive any external funding

## Ethics approval and consent to participate:

Not applicable

## Consent for publication:

Not applicable.

## Figures and Tables

**Table 1:** Score results between obesity and periodontitis-related proteins identified in the network interactions

**Case Number**	**Gene ID**	**Cluster**	**Distance**
1	HP	3	10.04
2	IGKV1D-33	3	10.24
3	LTF	3	0
4	IGLL5	1	13.69
5	S100A9	1	13.76
6	CA1	2	12.29
7	IGL@	1	4.24
8	IL1RN	1	7.92
9	ALB	3	9.93
10	CSTB	4	0
11	PRDX5	1	8.55
12	PRDX6	1	9.41
13	ENO1	1	3.21
14	PGAM1	1	12.74
15	SERPINB9	1	8.68
16	ANXA1	2	0
17	GSTP1	1	0
18	CA1	2	6.44

**Table 2:** Core clusters of proteins

**Final Cluster Centers**				
	**Cluster**			
	1	2	3	4
Coverage	47.5	64.6	28.8	95

**Table 3:** ANOVA results of protein clusters

**ANOVA**						
	**Cluster**		**Error**		**F**	**Sig.**
	Mean Square	df	Mean Square	df		
Coverage	1496.127	3	53.852	14	27.78	0

**Table 4:** Details the identified proteins in the interaction between obesity and periodontitis

**Protein Symbol**	**Name**	**Description**
ENO1	Enolase	A key glycolytic enzyme involved in converting 2-phosphoglycerate to phosphoenolpyruvate.
PRDX5, 6	Peroxiredoxin5	These are antioxidant enzymes that play a role in reducing hydrogen peroxide and protecting cells from oxidative damage.
PGAM1	phosohoglycerate	Another glycolytic enzyme (phosphoglycerate mutase), which works closely with ENO1.
GSTP1	Glutathione s transferase	A glutathione S-transferase involved in detoxification and stress responses.
SERPIN	Serpin	Inhibitor of granzyme B, involved in immune surveillance and apoptosis.
CSTB	Cystatin B	A cysteine protease inhibitor, often involved in inflammation and proteolytic pathways.
S100A9	S100A9	A calcium-binding protein involved in inflammatory responses and immune cell recruitment.
ANXA1	Annexin	Known for its role in anti-inflammatory processes, modulating immune cell responses.
LTF	Lactotransferrin	Involved in iron binding and has antimicrobial properties.
FAIM2	FAs apoptotic inhibitory molecule 2	Associated with resistance to apoptosis (programmed cell death)
IGLL5	Lambda - like polypeptide 5.	Associated with immunoglobulin functions and B-cell development
